# Spouses' Experiences of Emotional and Existential Support When Caring for a Frail Partner Late in Life

**DOI:** 10.1111/opn.70028

**Published:** 2025-04-23

**Authors:** Helena Larsson, Anna‐Karin Edberg, Kerstin Blomqvist

**Affiliations:** ^1^ Faculty of Health Sciences Kristianstad University Kristianstad Sweden; ^2^ The Research Platform for Collaboration for Health Kristianstad University Kristianstad Sweden

**Keywords:** content analysis, emotional concerns, existential concerns, focus group, partner late in life, spouse, support

## Abstract

**Introduction:**

The ability to care for a frail older partner late in life often entails the need for support and help from others, a need that sometimes can go unmet. Exploring spouses' views of emotional and existential support can guide further development of supportive structures, which in turn can promote family caregivers' existential health and well‐being. Therefore, the aim of this study was to explore what spouses experience as supportive of their emotional and existential concerns when caring, or after having cared for, a frail partner late in life.

**Methods:**

The study was explorative and based on multistage focus group interviews with older spouses (*n* = 10) divided in two groups who met three times each. The data were analysed using conventional content analysis. The checklist ‘Consolidated criteria for reporting qualitative research’ (COREQ) was followed when presenting the study.

**Results:**

The spouses described the importance of an atmosphere in which being sad was allowed for as much time as needed; it was safe to share experiences together with others, they could receive compassion and comfort from others, and they were free to feel hope, let their previous life go and dare to think of their future.

**Conclusion:**

Providing emotional and existential support creates an atmosphere that allows older spouses to reflect together with others, listening to their own and others' thoughts, and thus be able to put feelings and experiences into words. A suggestion for organising such support considers the physical, social, personal and spiritual dimensions of people's lifeworlds.

**Implications for Practice:**

Nursing interventions aimed at improving emotional and existential support for older spouses should primarily target transitional phases in life and focus on relational aspects.

**Summary**

- What does this research add to existing knowledge in gerontology?
  ○ An understanding of the physical, social, personal and spiritual dimensions of people's lifeworlds for facilitating emotional and existential support to older spouses.
  ○ The importance of an atmosphere where spouses can be sad for as much time as needed, feel free to hope, let their previous life go and dare to think of their future.
- What are the implications of this new knowledge for nursing care for and with older adults?
  ○ Nursing interventions aiming at improving emotional and existential support for older spouses are important to enable them to remain supportive of their partners as well as maintain their own well‐being.
  ○ Interventions focusing on emotional and existential support for spouses caring for a frail partner late in life are essential in transitional phases and should focus on relational aspects.
- How could the findings be used to influence practice, education, research, and policy?
  ○ Knowledge about spouses' emotional and existential needs when caring for a frail partner late in life can be used as a base for the development of future intervention studies.
  ○ Organising forums that focus on narrations regarding the current life of spouses caring for a frail partner can be a way to provide emotional and existential support.

## 1 Introduction

Caring for a frail partner late in life often entails unmet needs (Larsson et al. 2020). Providing help and support for another person can be satisfying, but it can also be burdensome and lead to ill health (Greenwood and Smith 2016), exhaustion, stress and isolation (Stoltz et al. 2004). During ageing, existential concerns become increasingly central and are often linked to the last phase in life (van Wijngaarden et al. 2015) and to feelings of existential loneliness and meaninglessness (Sjöberg et al. 2018). Supporting a partner late in life, support and help from others are therefore needed. For a person to feel whole, their physical, social, personal and spiritual needs must be addressed (Larsson et al. 2020). Although the need for support is often highlighted (Larsson et al. 2020; Greenwood and Smith 2016; Stoltz et al. 2004; Hellström et al. 2017), there is a need for further understanding of holistic support where emotional and existential support is addressed.

Existential concerns are often referred to as questions related to our existence as human beings. Yalom (1980) describes *the ultimate concerns* that deal with the finality of life, the need for freedom and togetherness, and the search for meaning (Yalom 1980). Confrontation with these core aspects of human life often prompts thoughts about the meaning and value in life and can result in existential distress (Grech and Marks 2017). In addition, when a person's existence is threatened, existential suffering may occur (Sand and Strang 2013). The results from a study in Sweden with spouses caring for a frail partner late in life revealed existential challenges, for example, when the spouses experienced inner struggles, felt forced to make difficult choices and confronted the permanent loss of their partner (Larsson et al. 2020). Having to face the death of a partner means living in the presence of death, a phase when the uncertainty and anxiety of family caregivers needs to be acknowledged (Melin‐Johansson et al. 2012). A study based on interviews with family caregivers (75 years and older) who cared for someone close to them showed that they often found it difficult when they felt lonely and had their own health problems, but at the same time, they found caregiving to be satisfying (Greenwood and Smith 2016). Neri et al. (2012) showed that 65% of older family caregivers experienced both physical and emotional exhaustion when caring for someone close to them (Neri et al. 2012). Several studies have explored family caregivers' need for support in specific contexts—for example, in relation to specialised palliative care, where family caregivers express a need for support, especially concerning their own emotional health (Norinder et al. 2021). Earlier studies have also shown that spouses are in a vulnerable situation when in limbo between ‘we, together’ and ‘me, on my own’ and that more research is needed to understand how to best provide support for emotional and existential concerns (Larsson et al. 2020; Greenwood et al. 2019; Fowler et al. 2022). Studies often highlight that family caregivers have continuous needs that are not always noticed, which seems to be one reason for failing support (Greenwood et al. 2019; Fowler et al. 2022; Anker‐Hansen et al. 2018; Plöthner et al. 2019; Oh and Han 2019; Førsund et al. 2015; Pozzebon et al. 2016; Munkejord et al. 2020). A meta‐study of six systematic reviews compiled family caregivers' needs when caring for an older person with dementia. Results indicated that the family caregivers wanted support in 20 different areas—for example, emotional concerns and the transition when their partner had to move from home to a nursing home (Atoyebi et al. 2022). Caring for a spouse late in life thus entails emotional and existential challenges that need to be considered in the development of supportive structures.

Even though it is stated that support from the healthcare services should be provided to family caregivers, it is sometimes unclear what this support should involve (Stoltz 2006; The National Board of Health and Welfare 2001). To define support in relation to coping with a vulnerable and stressful situation, Cohen's (2004) description of social support can be used. According to Cohen, social support involves instrumental support, which comprises practical support with daily living; informational support, which means providing knowledge and guidance; and emotional support which implies showing compassion and mutuality (Cohen 2004). In addition, Langford et al. (1997) describe appraisal support as providing confirmation to strengthen another person's self‐value (Langford et al. 1997). In addition to these types of support, existential support has been described by Strang et al. (2001) in a study involving patients with life‐threatening illness, and their spouse and nurses. The result showed that the three groups had different perceptions of what constitutes existential support. For example, although the nurses related existential support to religion, the patients defined this support as a holistic way of thinking. In addition, the spouses related existential support to feeling seen. However, the common finding was that they all described existential support as being provided through genuine conversations (Strang et al. 2001). A further exploration of spouses' views of emotional and existential support can guide further development of supportive structures, which in turn can promote family caregivers' existential health and well‐being. Therefore, the aim of this study was to explore what spouses experience as supportive of their emotional and existential concerns when caring, or after having cared for, a frail partner late in life.

## 2 Material and Methods

### 2.1 Design

The study is part of the LONE study (RR2‐10.2196/1307) (Edberg and Bolmsjö 2019). In one of the studies, spouses who cared for a frail older partner were interviewed about existential loneliness (Larsson et al. 2020). During those interviews, the spouses also reflected on their need for support, and these narrations were analysed and are presented in the current study. The study was qualitative and explorative and was based on multistage focus group interviews (Hummelvoll 2008) with 10 spouses divided into two groups who met three times each. Repeatedly meeting the same group enabled a deep, reflective dialogue where it was possible to stay in the narratives shared between the participants. The data from the focus group interviews were analysed using conventional content analysis (Hsieh and Shannon 2005). The checklist ‘Consolidated criteria for reporting qualitative research’ (COREQ) (Tong et al. 2007) was followed when presenting the study.

### 2.2 Setting

In Sweden, the care of older people is mainly the responsibility of the 290 municipalities and is primarily funded by taxes. Relatives who provide care to a person at home are entitled to support under Swedish law. Support can include health care in the home, daily activities organised by the municipality or part‐time care at a nursing home. The study was conducted in one urban and one rural municipality in southern Sweden. The concept ‘frail’ was defined, both in the LONE study (Edberg and Bolmsjö 2019) and in the current study, as being 75 years or older, late in life and in need of long‐term health care.

### 2.3 Procedure

A family care advisor employed by the municipality was contacted and informed about the study and acted as the link between the first author and potential participants. Inclusion criteria were having experiences of living with and caring for a frail partner late in life, viewing themselves as a longstanding couple and having, or having had, the primary responsibility for their partner. To achieve a variety of experiences, we strove to include both men and women, those who lived together and those who recently had become a widow or a widower. The family care advisor asked spouses for permission to communicate their name and telephone number to the researcher. Spouses who were interested received a letter with information about the aim of the study, voluntariness and confidentiality. It was important that the group was of reasonable size, but not too large, partly to give everyone the opportunity to talk and partly, given the nature of the topic, to provide a safe atmosphere and an opportunity for trustful conversations. Therefore, the goal was for each focus group to consist of four to six participants (Hummelvoll 2008; Krueger and Casey 2015). In total, the contact information for 15 people was provided, 10 of whom agreed to participate.

### 2.4 Participants

The participants were 10 spouses—five men and five women—who were 67–89 years old (median age: 79.5 years). For further demographics of the participants, see Table 1.

**TABLE 1:** Demographics of the participants.

Spouses	*n* = 10
Women/men	5/5
Widow/widower, median (range)	3/2, 2 years (1–4 years)
Age, median (range)	79.5 years (67–89 years)
**Partner's care context** ^a^	
Municipal home care/service	6
Residential care	5
Living/lived at home	5
Specialised palliative care	3
**Partner's main concern**	
Dementia	6
Cancer	3
Other	1
Lived in the same municipality, median (range)	47 years (5–86 years)
**If something happens, do you have anyone to contact?**	
Yes	10
**Do/did you have anyone who relieves/d you?**	
Yes	5
No need	4
No	1
Lived together as a couple, median (range)	51.5 years (46–65 years)

^a^ Multiple caregivers possible.

The participants were divided into two groups depending on whether they lived together with their partner or whether they were a widow/widower. Our aim was to create a reflective dialogue and interaction in each group. According to Hummelvoll (2008), a prerequisite is that the participants have similar situations and experiences to share and discuss. Therefore, the first group consisted of five spouses who lived together with their partner, whereas the second group consisted of five spouses whose partner had recently died.

### 2.5 Data Collection

In total, six focus group interviews were conducted, that is, two groups who met three times, respectively. Multistage focus group interviews are characterised by the same group meeting several times over a prolonged period, which provides the opportunity to achieve a deep, reflective dialogue (Hummelvoll 2008). Conducting the interviews in groups allowed for reflections together on a specific topic, and the group promoted dialogue and interaction (Krueger and Casey 2015). The interviews took place between August and October 2018. Each interview lasted about 2 h and was conducted in a room provided by the municipality. There was a 2‐week gap between Interviews 1 and 2, and a 3‐week gap between Interviews 2 and 3. The time and day for each interview were decided together with the participants. Both focus groups were kept intact through all three interviews, and all participants, except for one participant in each group, took part in all focus group interviews. Each focus group interview was recorded and transcribed verbatim.

**FIGURE 1:**
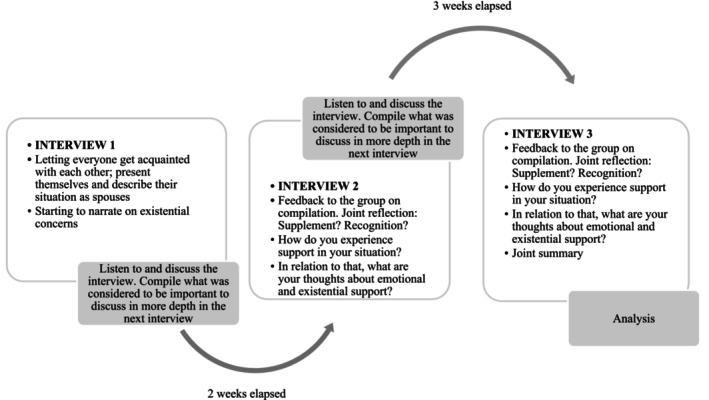
Procedure during the data collection.

During the interviews, the first author acted as a moderator leading the conversations, whereas the last author acted as an observer, took notes, observing the atmosphere, drawing conclusions and asking follow‐up questions. According to Hummelvoll (2008), the first interview should be devoted to creating a good atmosphere in the group. This was done by letting everyone in the group present themselves to each other and describe their situation as spouses. Thereafter, the interview proceeded by talking about needs and support. Questions the participants were asked to reflect upon were: How do you experience support in your situation? In relation to that, what are your thoughts about emotional and existential support? Probing questions were used to provide more depth in the interviews, like: How did you experience the situation? How would you like it to be? In the group with widows and widowers, participants were asked to reflect upon how they experienced support when their partner lived, in relation to how it was now. In both groups, participants were encouraged to talk about their experiences and to reflect on both their own and each other's narrations. The second and third interviews were introduced with a compilation from the previous interview and started with a joint reflection on the conclusion. For a description of the data collection procedure, see Figure 1.

### 2.6 Analysis

Data were analysed using conventional content analysis, often used when existing literature is lacking (Hsieh and Shannon 2005). A few days after each interview, the first and the last author met to listen to and discuss the interview, and then compile what was considered to be important to discuss in more depth in the next interview. When all six interviews—that is, three interviews in each group—were completed, analysis of the transcribed interview texts (in total, 525 pages) was started. In the first step, the interview text was read as a whole unit by the first and last author. When something emerged related to the aim of the study, this was highlighted, and notes were made about the first impressions, respectively. Thereafter, the first and the last author met to discuss their impressions and highlighted texts. In the second step, the highlighted text (‘meaning units’) was transferred to a separate document. The meaning units were given a code—that is, a short description of the essence of the meaning unit, still close to the interview transcript—for example, ‘confirmation in the loss’ or ‘time as a prerequisite’ or ‘worried, mourning’. In the third step, the codes were clustered, that is, sorted together depending on how they were linked to each other. For example, all codes concerning future and hope were clustered together and codes concerning time were placed together. When the first author had clustered all the codes in different groups, the first and the last author met to discuss this sorting. The sorting was then discussed between the three authors, resulting in four categories, and finally verified in relation to the interview transcripts. For an example of the analysis process, see Table 2.

**TABLE 2:** Example of meaning unit, code and category.

Meaning unit	Code	Category
I: The quality time it [meeting] gives a deeper comfort… and can probably be a relief, I think. H: So, it is important to find… yes, those gatherings and situations… where you find deeper meaning, where you can receive comfort, yes, and it is pure chance sometimes. Focus group 2, meeting 3	Gatherings with deeper meaning	Having a space where it is safe to share experiences together with others
E: … we have our best friends 5 minutes' walk from where we live and it is nice to have that feeling, that you can go there and talk whenever you want. Focus group 1, meeting 2	Have close friends nearby	
A: …those meetings and the things they [municipality] have organised, that you are more or less… in the same situation, but still that you have a certain sense of togetherness and… that it is easier to talk then, than to talk to someone who has no idea of what you are really going through. C: … and groups like this… it is just right, you should not be too many… because then you become more reserved perhaps towards each other; you become… more open if you feel togetherness. Focus group 1, meeting 3	To feel togetherness, meeting others who are in a similar situation	

### 2.7 Ethical Considerations

The study was approved by the Ethical Review Board, Sweden (2014/652; 2018/422). The first interview in both groups started with information about ethical issues, such as voluntariness and confidentiality. All participants signed an informed consent document. Thereafter, all three interviews in each group started with a reminder of voluntariness and the possibility to withdraw without explanation. If any participant expressed a need for further dialogue, the researchers had already made preparations to be able to share contact information with someone to talk to, for example, a family care advisor.

### 2.8 Trustworthiness

The trustworthiness of the present study has been considered according to dependability, credibility and confirmability (Shenton 2004). A measure for a study's dependability is the sample. At first glance, 10 spouses may seem like a small number of participants. However, in total, six focus groups interviews were conducted, which generated 12 h of recorded data and 525 pages of transcribed interview text. Variation in the sample is another measure for dependability, and earlier studies have shown that existential concerns and the need for support are prominent in transitional phases, for example, when deciding to move a partner into a nursing home (Larsson et al. 2020; Greenwood et al. 2019). Therefore, we strove to reach not only spouses who had care responsibilities at that time but also those whose partner had died. During the data collection period of 5 weeks, two of the participants belonging to the first group became widows/widowers. Despite the trauma of their partner's death, those participants chose to take part in the next focus group interview because they saw the conversations as important. That they chose to come can be seen as an expression of the importance of the study and that the atmosphere in the group was permissive and promoted deeper conversations. In addition, it enabled us to capture the need for support in emotional and existential concerns over time when caring for a frail partner late in life. It is also noteworthy that the age span of the spouses encompassed 22 years (67–89 years). This is foremost a strength, as it increases variation in the sample and the spouses' experiences, something to strive for in qualitative studies (Merriam and Tisdell 2016). Regarding credibility, the authors decided after careful consideration to collect data through multistage focus group interviews (Hummelvoll 2008). According to Krueger and Casey (2015), focus group interviews facilitate discussions and a broader perspective on a topic as participants can build on each other's thoughts and ideas, which can lead to deeper insights and new ideas that might not have emerged in an individual interview (Krueger and Casey 2015). In addition, Hummelvoll (2008) argues that repeated sessions promote a calmer atmosphere than if ‘all things’ had to be said during one interview. Furthermore, the same group was interviewed repeatedly over a prolonged period to facilitate reflections and dialogue and thus achieve depth in the interviews (Hummelvoll 2008). In addition, the researchers conducting the interviews and the analysis are familiar with the topic of emotional and existential concerns, such as existential loneliness and meaning in life. Carefully, and in accordance with Hummelvoll's (2008) recommendations, we planned how many interviews it was reasonable to demand from the participants. When meeting the group, we decided together when to meet the next time, and our joint decision during the process was that three meetings provided sufficient space for in‐depth discussions. Concerning confirmability, the authors have vast knowledge regarding research in the field of nursing, older adults, palliative care and existential phenomena. To safeguard confirmability, we have striven to be aware of our preunderstanding by continuously discussing our assumptions during data collection and later during analysis. The results are presented with quotations from the interviews to make it possible for the reader to verify the analysis. Each step in the analysis is described in text, tables and a figure.

## 3 Results

The spouses' experiences of support regarding their emotional and existential concerns showed the importance of an atmosphere in which (1) being sad is allowed for as much time as needed, (2) it is safe to share experiences together with others, (3) they can receive compassion and comfort from others, and (4) they feel free to have hope, let their past life go and dare to think of their future.

When presenting the results, quotes are used from the interviews carried out in the two focus groups (stated as Focus groups 1 and 2). To identify whether the quotation comes from meetings 1, 2 or 3, these numbers are provided in the text. Letters are used to indicate the different spouses speaking. In Focus group 1, the spouses are coded with the letters A, B, C, D and E. In Focus group 2, the spouses are coded with the letters F, G, H, I and J.

### 3.1 Being Sad Is Allowed for as Much Time as Needed

The spouses expressed a deep sadness in their unwanted situation but also stated that it was supportive when they felt that being sad was allowed. They missed and longed for their partner and expressed how they felt lost and insecure and how their life had been ‘put on hold’ as they were going through a transition in life. Their partner was being cared for in a nursing home instead of at home with them, or their partner had died, and they felt forsaken. The spouses described how they felt worried and anxious but also how they allowed themselves to feel that way. It was supportive when they felt allowed to be sad together with friends or family. On several occasions, they added words like ‘I have reason’ as a way of showing that they allowed themselves to be sad.

J: …nothing is really good yet…I am allowed to be worried, I tell myself that, that it is no wonder that I am…am as I say anxious and worried, because I have reason… I: …and I have mourned it over time… H: …it is so to say…a life crisis, that is the kind of thing we are talking about…. (Focus group 2, meeting 3)

The spouses highlighted that each person needed different amounts of time and that no one other than themselves could know when they were ready to move on. The time of grief was described as coming and going in that they had moments when they felt sad but also moments when everyday life was going well. They spoke of the importance of an atmosphere in which the people around them allowed them to have *time* to think, *time* to be alone, and *time* to grow accustomed to their loss.

E: Everyone needs a bit of time alone…//…I have to be able to think for myself…//…be out in my carpentry and work and so on, then I feel good…you have to have time… A: …//… That it is so important to take time to go back in your life and remember situations and sometimes understand them in a different way than you did when you were in the situation… E: …//… Yes, to have time…. (Focus group 1, meeting 2)

### 3.2 Having a Space Where It Is Safe to Share Experiences Together With Others

The spouses described that it was supportive to feel that they were not alone. They felt supported when they felt safe enough to share what they had been through together with others. Finding a safe space to share and vent about the situation involved discovering a setting in which they felt connected and they belonged—a space where they were allowed to show their feelings and talk about how their life was now and how it had been before. The narrations covered the importance of having family or friends physically nearby, such as living close enough to easily visit each other and to talk for a while. It was also about having someone who was easy to call when grief came over them. The narrations covered the importance of meeting others who were in a similar situation, for example, through organised gatherings at municipality centres or pensioner organisations, by the Red Cross or at churches. It was important to have a setting in which it was possible to share difficult experiences. This safe space was described using the metaphor of having a ‘safety valve’ to let out the bereavement and sorrow, instead of carrying the burden alone.

C:…the more you talk about something, the more you let it out…so you do not carry it inside you in the same way if you have someone to share it with…// A: …we can call it a kind of safety valve when we let it out…the risk is otherwise, if you do not have someone to share it with, that you carry it inside you and that it becomes a big lump. (Focus group 1, meeting 3)

Being part of an organised conversation group was also thought of as a space where it was possible to share their feelings together with others. The spouses appreciated when the conversation group was not made up of just any people but of people who had similar experiences as them, where they could recognise each other's situations and feelings. Meeting others with similar experiences helped them to feel a sense of belonging.

I: It is the same for all of us, we are in the same boat… G: Yes… I: You can take comfort in that, that is just the way it is. F: …for me it felt difficult in the winter …, but then the healthcare service had arranged this group, the support group that started in mid‐February…Then it became a bit easier, then I met people in the same situation…well…like a dozen others. (Focus group 2, meeting 1)

It was important to talk repeatedly and share the experiences both of when their partner lived and also after their partner's death. The spouses described the importance of others talking about what had happened as a confirmation of their own experiences of the situation.

H: …to be able to tell you a hundred times…how it is…how it was…that's the only way to move on…//…so when this pops up and to be able to talk to my daughter …//…so nice to be able to do that…yes, that others… I: …not only that you talk about the death, but that others do it too. (Focus group 2, meeting 3)

### 3.3 Receiving Compassion and Comfort From Others

The spouses said it was supportive when people in their vicinity showed compassion for them and their partner. It comforted them and gave them a feeling of being valued and important. The need for compassion and comfort from others was the most prominent need expressed by the spouses. To have value and to matter to someone else gave meaning to their life. It was important to remember the partner as they were, even if illness or changed personality had made them different, or if their partner had died. Experiencing compassion was about feeling how staff, for example, at the nursing home, could understand what was important for them as a couple, like sharing evening meals together even though it was too late for visiting, or arranging for them to get out and walk together even though it meant extra work for the staff. It was also about how spouses felt well when they knew their partner was receiving good care and attention. Compassion was a help and support, providing comfort and a way to find meaning in the difficult situation.

A: …the staff are good there, I believe; they do not complain as much about him there, as they did at…and it was that he wanted to go out and walk and they found that hard, having to go out and walk with him. But they are happy to do that here…I am glad…it makes me happy… [Interviewer: does it make you feel good to know that he is well?] …yes…yes, it really does. B: Yes, but you do. He has had a great time up there; they have all been so nice…//…they will miss him too. (Focus group 1, meeting 3)

The spouses described themselves as ordinary people who needed the same things as people without grief, like being invited home for coffee, and how this was particularly important when they were in a vulnerable and lonely situation.

I: …and before you come to invite friends to your home and you would have liked to be invited yourself, I felt, to the neighbours for some coffee and so, you are still an ordinary person, although you are grieving…//… J: …you need it very much then, you need a lot of compassion and comfort. (Focus group 2, meeting 2)

Turing to spirituality for support and searching for meaning were described as comforting. This involved believing in a higher power, praying or reading the Bible. It was also about spending time in nature, listening to music or reading books.

F: …for many it is a comfort, that is how it is…. H: …I can surrender and believe in a divine power…//…having a faith and being anchored in it can be something that helps us humans…//… F: [hum]…//… I: …but not all have a faith… F: …but it can be nature for someone else… H: …and literature for others…. (Focus group 2, meeting 3)

It was supportive if their partner's life ended easily so that spouses could look back on that time with fond memories. They expressed how staff were compassionate and supportive when making their partner's late life memorable; one way to cope with the loss of their partner was to consciously direct their thoughts towards fond memories. Even after their partner's death, they continued to think about him or her and how it felt supportive and comforting to do so. One way to feel at peace with the situation was by remembering their partner and talking to them. They described how they saw, spoke with and smelt the fragrance of their partner even though they knew their partner was not alive, and how it gave them comfort.

H: …surprising and sometimes it feels a bit comforting…//…comforting, yes, when not everything is cut off…//…I meet my husband many, many times in the borderline between waking and dreaming…//…I do hear him…// J: …mm…I pretty much dream every night…//…about my husband in some context and he is always healthy. (Focus group 2, meeting 3)

### 3.4 Feeling Free to Hope, Let Their Past Life Go, and Dare to Think of Their Future

The spouses described how they wanted to look forward and how they hoped that their situation would improve and their sadness would ease. Feeling hope and dare to think ahead were expressed as a way to feel free instead of limited. Phrases, such as ‘dare, at the risk of disappointment’, were used, and they recognised how there was much to be gained if they dared to think ahead. Thinking forward and letting go of the past were described as a huge step, for which courage was needed.

I: …You have so much to gain, maybe a little to lose, you never know, but you have a lot to gain, I think…if you dare to take the step, but it is a huge step, that is it…you can be disappointed, you never know…but that is how life is, I think, at any age…. G: Yes, it is like that. I: …you have to take the opportunity to let go…. (Focus group 2, meeting 2)

After the loss of the partner, one strategy was to get through the difficulty *for the sake of* their partner. Expressions, such as ‘life must go on’, were used as a statement for how there was no other way to get through the sadness. They needed to hope for a future even if it was without their partner.

J: You cannot wait… F: well, I felt that way too, you have to…well, life goes on…//… I: *You must try*. That is what they would have wanted, our partners. Not just sitting alone and feeling down…. (Focus group 2, meeting 2)

## 4 Discussion

This study aimed to explore what spouses experienced as supportive of their emotional and existential concerns when caring for, or after having cared for, a frail partner late in life. The results showed the importance of an atmosphere where (1) they were allowed to be sad for as much time as needed, (2) it was safe to share experiences together with others, (3) they could receive compassion and comfort from others, and (4) they could feel free to have hope, let their past life go and dare to think of their future. These results can be understood in light of the lifeworld perspective (Lebenswelt), which has its roots in existential philosophy and assumes that people are in a constant transition through life (Sartre 1947/2007; Jaspers 1971/1995). The existential psychologist Emmy van Deurzen (van Deurzen 2012) has embraced the lifeworld perspective, which explains wholeness by considering people's lifeworld in four dimensions—the physical ‘Umwelt’, social ‘Mitwelt’, personal ‘Eigenwelt’ and spiritual ‘Überwelt’. For a person to feel whole, the four dimensions of the lifeworld need to be addressed (van Deurzen 2012). Being aware of the four dimensions can facilitate the development of structured support focusing on the wholeness of a person, where meeting the emotional and existential needs of spouses caring for a frail partner late in life is essential.

The results show that an allowing and inclusive atmosphere is key to feeling supported in emotional and existential concerns. The spouses talked about an allowing atmosphere where they were free to tell their story on repeated occasions and to have someone with the patience to listen without judgement or trying to console. In line with previous research from Switzerland (Ris et al. 2019) and the United States (Ermer and Proulx 2020), this study shows that an inclusive and welcoming atmosphere promotes well‐being for people who care for a family member late in life. The social dimension in a person's lifeworld involves how people relate to others as they interact with those around them (van Deurzen 2012). This was expressed by the spouses, who described that it was supportive to feel that they were not alone, and that they needed to feel that it was safe to share experiences together with others. Tillich (1952/2000) states that everyone is in a constant state of tension between oneself and the world, and according to Buber (1923/2003), it is in our relationships that we exist. Being anchored to something, such as a significant place, or to someone, such as a partner, is about experiencing an encounter. Buber (1923/2003) argues that we can relate to and experience an encounter with nature, where the relationship is wordless; with people, where the relationship can take the form of language; and with the spiritual world, where the relationship comprises thoughts (Buber 1923/2003). Creating a relational anchorage to something or someone could thus mean providing the conditions for encounters not only with other people but also with nature and a spiritual dimension, thus providing emotional and existential support. Healthcare professionals, such as nurses thus need to be aware of and pay attention to the importance of the relational aspect in their encounters with spouses who care for a frail partner late in life.

Another key to feeling supported in emotional and existential concerns is connected to the person's own ability to strive for meaning. The results show that the spouses strive to feel that life is meaningful and to find a new way to move forward into the future without their partner—to ‘let their past life go’. A recent study shows that loneliness is a common feeling when entering a new phase in life without a partner and highlights the importance of support from society, family and friends (Van Hout et al. 2020). However, the present study also reveals the importance of support in concerns about the future. The spiritual dimension relates to the unknown and how people view meaning in life and think of their future (van Deurzen 2012), which is related to the spouses' endeavours to feel free to have hope, let their past life go and dare to think of their future. The spouses strive to gain control over their new situation, to feel free instead of limited and to do what they want to in *their own way*. Jaspers (1971/1995) argues that throughout life people are in constant transition between past, present and future. Being in transition involves existential concerns, such as the search for meaning and freedom (Jaspers 1971/1995). The search for meaning is described as a force in life, and although meaning changes throughout life, the quest for it never ceases (Frankl 1946/2008). Frankl (1946/2008) describes that meaning in life can be achieved by performing an action, such as the spouses spending time with family. In addition, Frankl refers to how meaning can be achieved by feeling the value of experiencing something, such as when the spouses talk about how important it is to meet other people in a similar situation as their own; that is, they give each other meaning by supporting each other. Furthermore, this study shows that an important question is how can healthcare professionals support those who do not find meaning in life when losing a life partner? A qualitative study with older women shows the importance of recognising sources of inner strength and how togetherness, creativity and security can promote finding inner strength (Boman et al. 2015). Such findings are well in line with the results of this study. The present study also reveals that several of the spouses talked about the importance of time, that is, that time makes ‘the difficult’ bearable. Mourning takes a different amount of time for different people, and sadness often comes and goes over a prolonged period (Costello and Kendrick 2000). The physical dimension of a person's lifeworld concerns not only people's attitudes to their bodily needs but also their needs in relation to the pace and space of life (van Deurzen 2012), which was expressed by the spouses as the importance of feeling allowed to be sad for *as much time as they needed*. Acceptance of the past can make the present acceptable and give confidence for the future (van Deurzen 2012). Research shows that spouses of older people suffering from cancer go through grief as a process with different phases and that the phases vary in length and intensity (Van Hout et al. 2020). Jaspers (1971/1995) describes transitional phases in life as a struggle to ‘get beyond every horizon’; that is, the spouses strive to find a new way to cope in everyday life. Different circumstances can ease the process of getting through difficulties, such as belonging to a discussion group, whereas other circumstances can hinder the process, such as when existential needs are not acknowledged. Empowering circumstances that can help a person get through emotional and existential suffering might facilitate people in finding existential health (Melder 2011). To understand another person is a way of acting and thinking from a person‐centred approach, which means being intentional, facilitative and present (Williams and McCormack 2017). What enhances a person's existential health is individual; therefore, it is important that healthcare professionals are person‐centred to focus on each person's unique needs. The personal dimension concerns how people view themselves (van Deurzen 2012), which was seen when the spouses received compassion and comfort from others, thus experiencing that they had a value and mattered. A qualitative study from Sweden on what alleviated existential loneliness for frail older people shows that it is important to feel acknowledged by others and to feel that other people care (Sjöberg et al. 2019). This is in line with Buber's (1923/2003) description of relationships with the words ‘I’ ‘you’ and ‘it’. When relationships consist of meetings between ‘I’ and ‘you’ there are conditions for comforting encounters between people. A genuine meeting occurs when it is possible to share what is important (Buber 1923/2003). If we as humans do not have, or cannot maintain, relationships where genuine encounters occur with the possibility to share the essentials of life, the meaning in life can be lost. Therefore, it is vital for human existence to feel that there is someone who shows a genuine interest (Buber 1923/2003). When encountering older spouses who care for a frail partner in late life, it is therefore important to have the physical, social, personal and spiritual dimensions of a person's lifeworld in mind.

## 5 Limitations

A potential threat to the dependability of the study is that 6 years elapsed between data collection and publication of the results. However, according to Yalom (1980), the ultimate concerns in life, such as the search for meaning, freedom and togetherness, will never change. Regarding transferability, the fact that all participants were Swedish born and that the study was conducted in a Swedish health care context might limit transferability. However, the participants were of different ages, from two different municipalities, and their partners received care in diverse care contexts, which may strengthen the transferability of the results.

## 6 Conclusions

This study aimed to explore what spouses experienced as supportive of their emotional and existential concerns when caring, or after having cared for, a frail partner late in life. To provide emotional and existential support is to create an atmosphere that allows older spouses to reflect together with others, listening to their own and others' thoughts, and thus be able to put feelings and experiences into words. Such support should consider the physical, social, personal and spiritual dimensions of people's lifeworlds. What enhances a person's emotional and existential health is individual, so it is important to provide person‐centred care with a focus on each person's unique needs. Interventions including emotional and existential support should primarily target transitional phases in life and focus on relational aspects. Organising forums where narrations about life are at the centre can be a way to provide such support. For future research, there is a need for intervention studies with focus on organised support for spouses who care for a frail partner late in life. Such support could benefit many people, enabling spouses to remain supportive of their partners as well as maintaining their own well‐being.

## Author Contributions

H.L., K.B. and A.K.E. designed the study. H.L. and K.B. collected the data. H.L. and K.B. had the main responsibility for the analysis while A.K.E. confirmed the analysis. H.L. had the main responsibility for drafting the manuscript, but all authors contributed substantially to the text and approved the final version of the paper.

## Ethics Statement

The study was approved by the Ethical Review Board, Sweden (2014/652; 2018/422).

## Conflicts of Interest

The authors declare no conflicts of interest.

## Data Availability

The data generated and analysed during the current study are in Swedish and are not publicly available but are available from the corresponding author upon reasonable request.
